# A24 GENERALIZABILITY OF CROHN’S DISEASE RANDOMIZED CONTROLLED TRIALS COMPARED TO CLINICAL PRACTICE

**DOI:** 10.1093/jcag/gwab049.023

**Published:** 2022-02-21

**Authors:** T Chhibba, A Frolkis, C Ma

**Affiliations:** 1 Gastroenterology, University of Toronto, Toronto, ON, Canada; 2 University of Calgary, Calgary, AB, Canada

## Abstract

**Background:**

There are no curative medical therapies for Crohn’s disease (CD). However, multiple novel treatment options are currently being evaluated in randomized controlled trials (RCTs). Historically, CD populations enrolled in RCTs have not reflected the heterogeneity of patients observed in clinical practice due to strict inclusion/exclusion criteria and protocolized trial procedures do not reflect day-to-day care. These factors have raised concerns that results from pivotal RCTs required for drug approval are poorly generalizable.

**Aims:**

To assess the generalizability of CD RCTs, we evaluated the proportion of CD patients initiating ustekinumab or vedolizumab in clinical care who would have been eligible for enrolment in the corresponding phase 3 registrational induction trial, and the factors impacting trial eligibility.

**Methods:**

This is a retrospective cohort study using data from two ambulatory IBD clinics in Calgary, AB. Our study population included consecutive adult (>=18 years) patients with a confirmed diagnosis of CD, newly initiated on ustekinumab or vedolizumab between January 1, 2018 and January 1, 2020. We then applied the inclusion/exclusion criteria from the phase 3 GEMINI II/III and UNITI I/II induction trials to determine the proportion of patients who would have been trial-eligible. We then tabulated the indications for trial exclusion to determine the characteristics of patients who would not have been reflected in the trial population.

**Results:**

A total of 50 patients were included. The median age was 42.5 years. Most patients non-stricturing non-penetrating disease (44%, 22/50) and ileocolonic disease distribution (48%, 24/50). A total of 66% (33/50) would have been eligible for inclusion in GEMINI II/III and (30/50) 60% would be eligible for inclusion in UNITI I/II. The most common reasons for trial exclusion included extensive surgery (total colectomy/subtotal colectomy) with short bowel or ileostomy (n=12), surgery within 6 months of enrolment (n=3), multiple previous resections (n=2). Four patients were excluded based on intra-abdominal abscess. A total of 13 patients (26%) were temporarily ineligible due to recent biologic switch and would have required an 8-week washout prior to trial enrolment. Only two patients were excluded based on age (>80 years).

**Conclusions:**

Although most patients on ustekinumab or vedolizumab would have been eligible for the respective pivotal trials, patients who have complications of disease, extensive surgery, or altered anatomy are not reflected in current RCTs.

**Funding Agencies:**

None

